# The use of plasma exchange with albumin replacement in the management of Alzheimer’s disease: a scoping review

**DOI:** 10.3389/fneur.2024.1443132

**Published:** 2024-09-30

**Authors:** Yahveth Cantero-Fortiz, Mercè Boada

**Affiliations:** ^1^Ace Alzheimer Center Barcelona, Universitat Internacional de Catalunya, Barcelona, Spain; ^2^Networking Research Center on Neurodegenerative Diseases (CIBERNED), Instituto de Salud Carlos III, Madrid, Spain

**Keywords:** Alzheimer’s disease, plasma exchange, albumin replacement, cognitive function, amyloid-beta, neurodegenerative disorders

## Abstract

**Introduction:**

AD is a progressive neurodegenerative disorder causing significant cognitive decline and impaired daily functioning. Current treatments offer only modest relief, and many amyloid-targeting therapies have failed, prompting exploration of alternative approaches such as PE with albumin replacement.

**Objectives:**

This scoping review systematically maps the literature on PE with albumin replacement in AD management, focusing on outcomes, methodologies, and reported benefits and risks.

**Methods:**

A comprehensive search in PubMed, supplemented by reference scanning and hand-searching, identified studies involving PE with albumin replacement in AD patients. Data charting and critical appraisal were conducted using standardized tools.

**Results:**

Seven primary studies from the AMBAR (Alzheimer Management by Albumin Replacement) trial met the inclusion criteria, consistently reporting improvements in cognitive function, positive neuroimaging results, and favorable neuropsychiatric outcomes. For instance, one study found a significant slowing of cognitive decline (*p* < 0.05) among patients receiving PE with albumin replacement. Another study showed better preservation of hippocampal volume and improved brain perfusion metrics in the treatment group (*p* < 0.05). The intervention was generally well-tolerated with manageable side effects.

**Conclusion:**

PE with albumin replacement is a promising therapeutic approach for AD, warranting further investigation to confirm its efficacy and safety across broader settings.

**Scoping review registration:**

https://osf.io/v6dez/?view_only=1cd9637e7e0347d39713bf19aac0dfe8.

## Introduction

Alzheimer’s disease (AD) is a progressive neurodegenerative disorder characterized by significant cognitive decline and impaired daily functioning. It is the most common cause of dementia, affecting millions of individuals worldwide, and poses substantial social and economic burdens on patients, caregivers, and healthcare systems (1). The disease progression leads to loss of memory, language skills, and executive function, ultimately resulting in complete dependence on caregivers (2). Despite extensive research, the pathogenesis of AD is not fully understood, and current treatments provide only modest symptomatic relief without altering disease progression (3). The move towards a biological definition of AD, focusing on biomarkers like amyloid beta (Aβ) and tau, seeks to enhance diagnosis and treatment, but the disease’s mechanisms remain unclear (4).

The amyloid cascade hypothesis, which posits that the accumulation of Aβ plaques in the brain is a primary driver of AD pathology, has guided much of the therapeutic development in AD (5). Amyloid plaques are believed to disrupt cell-to-cell communication and activate immune responses that trigger inflammation and destroy neurons. On the other, hand maintaining Aβ balance between plasma and the brain is crucial, as highlighted by the peripheral versus central sink hypothesis. This hypothesis suggests that Aβ circulates between the brain and blood, and reducing peripheral Aβ could promote its clearance from the brain. Some Studies have proposed that lowering Aβ levels in the blood may create a ‘sink’ effect, encouraging its removal from the brain and potentially mitigating AD progression (6).

However, many therapies targeting amyloid plaques have failed to demonstrate significant clinical benefits, highlighting the need for novel treatment strategies (7, 8). Recent failures of high-profile amyloid-targeting drugs have shifted the focus toward alternative therapeutic approaches and combination therapies (9).

Plasma Exchange (PE) with albumin replacement is a potential therapeutic approach for AD. This method removes plasma containing pathological proteins like Aβ and replaces it with albumin. The rationale is that reducing peripheral Aβ levels may decrease its accumulation in the brain, as Aβ circulates between the brain and peripheral blood (10–14). Additionally, sequestering Aβ bound to plasma albumin through PE could induce an efflux of Aβ from the brain. The use of Aβ-free therapeutic albumin reinforces this mechanism, and albumin’s neuroprotective properties further facilitate Aβ removal (12, 15–19).

Recent studies, including the AMBAR (Alzheimer Management by Albumin Replacement) trial, have provided promising preliminary results suggesting that PE may be beneficial for AD patients. These studies have shown that PE can lead to improvements in cognitive function, delay in disease progression, and enhancements in quality of life (11, 12). The AMBAR trial specifically demonstrated significant cognitive improvements and reductions in amyloid levels, supporting the potential efficacy of this intervention (16).

Given the growing interest in PE with albumin replacement for AD, it is crucial to systematically evaluate the existing evidence to understand the intervention’s efficacy, safety, and potential mechanisms of action. This scoping review aims to map the current literature on this therapeutic approach, identify research gaps, and inform future research directions. By synthesizing the available evidence, we aim to provide a comprehensive overview that could guide clinical practice and future investigations in the field of AD treatment.

## Objectives

To systematically map the literature on PE with albumin replacement in the management of AD, focusing on:

- Different outcomes measured in these studies
- Various methodologies used
- Reported benefits and risks associated with the intervention

## Methods

The research team reviewed our protocol, developed using PRISMA guidelines (Supplementary Appendix 1). It was registered with the Open Science Framework on 05/22/2024 (OSF).

### Eligibility criteria

The eligibility criteria for this review were carefully selected to ensure the inclusion of relevant and high-quality studies. We included peer-reviewed studies that involved patients diagnosed with AD to focus on the specific target group affected by the intervention. The intervention of interest was PE with albumin replacement, and we included comparisons against standard treatment, placebo, or other interventions to assess the relative effectiveness. Outcomes of interest included cognitive function, brain structural changes, quality of life, and other related outcomes, which are critical measures of the intervention’s impact. We included randomized controlled trials (RCT), longitudinal studies, observational studies, and experimental studies to gather robust evidence from various high-quality study designs, published between the period of 2000–2024, written in English.

We excluded studies that did not involve AD to maintain focus on the relevant population, and studies not using PE or not involving albumin replacement to ensure the intervention of interest was being evaluated. Review articles, editorials, opinion pieces, and letters were excluded to focus on original research with primary data. Non-peer-reviewed articles were excluded to ensure the credibility and reliability of the included studies. Finally, studies not published in English were excluded due to practical constraints related to translation and ensuring the accuracy of non-English studies.

### Information sources

To identify potentially relevant documents, PubMed was searched from its inception to May 2024. The search strategy was drafted by an experienced member of our team and further refined through team discussion. The final search results were exported into EndNote, and duplicates were removed. The electronic database search was supplemented by scanning the reference lists of relevant reviews and hand-searching key journals. Additionally, gray literature sources and other databases such as Scopus and Web of Science were considered to ensure comprehensive coverage.

### Search strategy

The search strategy is shown in Supplementary Appendix 2. The search strategy included keywords and Medical Subject Headings (MeSH) terms related to AD, PE, and albumin replacement. This thorough search strategy ensures reproducibility and comprehensiveness in capturing all relevant studies.

### Study selection

Two independent reviewers conducted the selection process. Titles and abstracts were screened first, followed by a full-text review of selected studies to ensure they met the inclusion criteria. Both reviewers independently screened titles and abstracts, removing duplicates and excluding irrelevant studies. The reviewers then obtained and reviewed the full texts of relevant studies. Discrepancies were resolved through discussion between the reviewers, reaching a consensus without the need for a third reviewer. This process ensures rigorous and unbiased study selection.

### Data extraction and charting

A data-charting process was utilized by two reviewers to determine which variables to extract. The two reviewers independently charted the data, discussed the results, and resolved discrepancies through discussion or consultation with a third party. The data extraction form included variables such as study design, country, setting, participants (number, age, and sex distribution), interventions, comparisons, and outcomes. This robust and comprehensive data extraction process ensures that all relevant aspects of the studies are captured accurately.

### Critical appraisal of individual sources of evidence

To assess the methodological quality of the included clinical trials, we utilized the Cochrane Collaboration tool for assessing the risk of bias (RoB) (20). This tool evaluates seven key domains that can introduce bias into study results: random sequence generation, allocation concealment, blinding of participants and personnel, blinding of outcome assessment, incomplete outcome data, selective reporting, and other sources of bias. Each domain was assessed independently by two reviewers and rated as “low,” “high,” or “unclear” risk of bias, with detailed justifications provided for each rating. Discrepancies were resolved through discussion between the reviewers, and if necessary, consultation with a third reviewer was sought. This rigorous appraisal process ensured that our review provided a reliable and comprehensive synthesis of the existing evidence on PE with albumin replacement in managing AD.

### Synthesis of results

The synthesis of evidence addressed the research questions by summarizing findings in a narrative format, supported by tables. Studies were grouped by outcomes such as cognitive function, quality of life, and safety. Each group was summarized in terms of settings, populations, study designs, and measures used. Broad findings were highlighted to provide an overview of the evidence. Tables detailed study characteristics and results, offering a clear comparison of different studies. A visual representation was provided as a flowchart found in Supplementary Appendix 3. This approach ensured a comprehensive and clear presentation of evidence on PE with albumin replacement for AD.

## Results

### General characteristics of the included studies

In our initial search, we identified 39 articles, which were screened by title and abstract. From these, 14 articles were selected for full-text screening, resulting in 7 studies that met our inclusion criteria (10, 11, 15–19) (Supplementary Appendixes 3, 6). These studies focus on the use of PE with albumin replacement in patients with AD. The included articles are part of the AMBAR project, an RCT involving patients with mild to moderate AD, predominantly conducted in Spain. The total number of unique patients across these studies is 347, with some studies analyzing specific subpopulations within this main cohort. Thus, the same patients are often included across different studies, focusing on various aspects of the treatment outcomes.

The settings for these studies were clinical and hospital environments, providing a controlled setting for the interventions. The studies were conducted by two main research groups, Boada et al. and Cuberas-Borrós et al. The articles varied in their specific focus areas, including neuroimaging outcomes, cognitive function, and quality of life assessments. The study duration ranged from 21 weeks of treatment with a 6-month follow-up to detailed longitudinal analyses (Supplementary Appendix 4).

### Risk of bias assessment

The ROB assessment using the Cochrane Collaboration tool (20) revealed that all included articles presented a low ROB in the majority of the evaluated domains. Specifically, the study demonstrates adequate random sequence generation and allocation concealment, minimizing selection bias. Additionally, the blinding of participants, personnel, and outcome assessors was properly conducted across all studies, reducing the risk of performance and detection bias. It is important to note that this consistent low ROB is likely because all the articles belong to the same study (AMBAR trial). The studies managed incomplete outcome data adequately and avoided selective reporting, minimizing the risk of attrition and reporting bias, respectively. However, the domain of other sources of bias was marked as unclear due to insufficient information provided in the articles to assess this aspect fully. Despite this, the consistent methodological rigor observed in these studies contributes to the robustness of the conclusions drawn regarding the efficacy and safety of PE with albumin replacement in the management of AD (Supplementary Appendix 5).

### Outcomes

#### Effectiveness

The effectiveness of PE with albumin replacement in patients with AD was a consistent finding across the AMBAR studies. Multiple sub-studies reported improvements in cognitive functions and quality of life. For instance, Boada et al. (10) observed significant cognitive improvements in patients undergoing PE compared to the control group, correlating with changes in cerebrospinal fluid and plasma Aβ concentrations. Boada et al. (11) reported a 30% reduction in cognitive decline (*p* < 0.05) among patients receiving the treatment, further supporting the potential effectiveness of PE with albumin.

#### Neuropsychological and quality-of-life assessments

The AMBAR studies included detailed neuropsychological and quality-of-life assessments. Boada et al. (11) reported significant improvements in neuropsychological evaluations, including memory, attention, and executive function, as well as enhanced quality of life measures. Patients receiving PE with albumin demonstrated a 20% improvement in memory and attention scores compared to the control group (*p* < 0.05). The study employed standardized tests such as the Mini-Mental State Examination (MMSE) and the Alzheimer’s Disease Assessment Scale-Cognitive Subscale (ADAS-Cog) which demonstrated clinical improvements in the treatment group. However, these improvements did not reach statistical significance (*p* > 0.05) over the 21-week treatment period plus 6 months of follow-up.

#### Neuroimaging results

Neuroimaging analyses provided robust evidence for the structural benefits of PE with albumin. Cuberas-Borrós et al. (15, 17) demonstrated positive effects on brain perfusion, hippocampal volume, and intracranial volume. These studies used techniques such as magnetic resonance imaging (MRI) to measure changes in brain structure. The findings indicated that patients in the treatment group had a 15% better preservation of hippocampal volume (*p* < 0.05) and a 10% improvement in brain perfusion metrics compared to controls.

#### Neuropsychiatric assessments

Neuropsychiatric assessments in the AMBAR studies indicated improvements in mood and behavioral symptoms. Boada et al. (11) highlighted that patients receiving PE showed reductions in neuropsychiatric symptoms such as agitation, depression, and anxiety, as measured by the Neuropsychiatric Inventory (NPI). The reductions were statistically significant (*p* < 0.05) compared to the control group, suggesting that the treatment may have beneficial effects on both cognitive and neuropsychiatric outcomes.

#### Safety and tolerability

The safety and tolerability of PE with albumin were consistently reported across the AMBAR studies. Boada et al. (19) confirmed the feasibility, safety, and tolerability of two modalities of PE with albumin in elderly patients, noting that the treatment was well-tolerated with manageable side effects. Reported adverse events included mild to moderate hypotension and fatigue, which were effectively managed with standard care protocols. This finding was echoed in other sub-studies, such as Boada et al. (10), which also highlighted the favorable safety profile of the treatment.

## Discussion

In this scoping review, we identified and synthesized seven studies from the AMBAR trial, including one primary study and six sub-studies, all addressing the use of PE with albumin replacement in managing AD. The primary objectives of this review were to evaluate the effectiveness, safety, and methodological rigor of this intervention. Our findings indicate significant improvements in cognitive function and quality of life, as well as positive neuroimaging and neuropsychiatric outcomes associated with PE therapy.

### Effectiveness and benefits

The AMBAR studies demonstrated significant improvements in cognitive functions and reductions in Aβ levels in cerebrospinal fluid and plasma (10, 11). These findings support the hypothesis that removing Aβ through PE can mitigate its neurotoxic effects, thereby improving cognitive outcomes. Neuroimaging results from Cuberas-Borrós et al. (15, 17) showed positive effects on brain perfusion and hippocampal volume, suggesting that PE might also confer structural benefits to the brain.

### Safety and tolerability

The safety and tolerability of PE with albumin were consistently affirmed across the AMBAR studies. Boada et al. confirmed the feasibility, safety, and tolerability of two modalities of PE with albumin in elderly patients, noting that the treatment was well-tolerated with manageable side effects (16, 19). This aspect is crucial for the potential clinical application of PE, as the target population often includes elderly individuals who may have comorbid conditions. Other studies have similarly reported that PE is generally safe with a low incidence of severe adverse events (18, 19, 21, 22).

### Quality of life

The AMBAR trial indicated that PE with albumin replacement has the potential to improve the QoL in patients with mild AD, as reported by the patients themselves. Although the improvements noted by caregivers were not consistently significant, a positive trend was observed (11). These findings underscore the importance of considering QoL as a key outcome in evaluating new treatments for AD, particularly in the early stages of the disease.

### Methodological strengths

The methodological strengths of the included studies lie primarily in their rigorous design and implementation. All included studies were part of the AMBAR trial, which utilized RCT methodologies, ensuring a high level of evidence. The consistent use of blinding for participants, personnel, and outcome assessors in clinical trials minimizes performance and detection biases, ensuring the integrity of the study outcomes (10).

In the context of PE for AD, various studies have demonstrated the effectiveness and comprehensive approach of this treatment modality. For instance, the AMBAR study highlights the impact of PE on clinical laboratory parameters, showing significant changes in coagulation and blood composition, which support the treatment’s safety and efficacy (19). Additionally, research on APP/PS1 mice indicates that therapeutic PE can reduce amyloid plaque load and preserve vascular health, suggesting a beneficial effect on AD pathology (23). Further studies emphasize the method’s ability to eliminate neurotoxic chemicals from the blood, offering a plausible mechanism for slowing disease progression (6, 13). The feasibility, safety, and tolerability of different PE modalities in elderly patients with AD have also been confirmed, providing detailed insights into the practical application of this treatment (18). Finally, improvements in the quality of life for AD patients undergoing PE with albumin replacement have been documented, underscoring the holistic benefits of this intervention (15–19).

### Limitations

Despite the promising results, there are several limitations to consider. All the sub-studies were conducted by a single research group, which may introduce bias. Despite the moderate sample size of 347 patients, all the Studies involved the same population issue which limits the generalizability of the findings. Furthermore, the follow-up periods, although adequate for initial assessments, may not capture long-term effects and potential late-emerging side effects of the treatment. The geographical limitation to Spain also raises questions about the applicability of the results to more diverse populations. Future studies should include diverse populations and larger sample sizes to validate these findings across different demographics and settings (22, 24). Combining PE with other therapeutic approaches could also be investigated to potentially enhance treatment outcomes (22, 25, 26).

policymakers, the findings of this review highlight the potential of PE with albumin replacement as an innovative approach to AD treatment, which may warrant further investment in research and clinical trials (21). Healthcare providers may consider these results when exploring new treatment options for patients with mild to moderate AD, focusing on the intervention’s demonstrated benefits and manageable safety profile (15–19, 27). Patients and their caregivers can find hope in the emerging evidence supporting PE as a means to improve cognitive function and quality of life in AD management (19, 23, 27–30).

suggest that PE with albumin replacement offers significant benefits for patients with AD, including improvements in cognitive function, quality of life, and neuropsychiatric outcomes, with a favorable safety profile. However, the limitations highlighted necessitate further research to confirm these benefits. Future studies should include more diverse populations and larger sample sizes to validate these findings across different demographics and settings. Long-term studies are needed to assess the durability of the treatment effects and to monitor any late-emerging side effects. Additionally, exploring the cost-effectiveness of PE with albumin replacement will be crucial for its broader clinical application. Combining PE with other therapeutic approaches could also be investigated to potentially enhance treatment outcomes (28–32). By addressing these areas, future research can build on the current evidence to establish a more comprehensive understanding of the efficacy and safety of PE with albumin replacement in managing AD. This could ultimately inform clinical practice and policy, improving management and outcomes for patients with AD (26–29).

### Limitations of the scoping review

Our scoping review has several limitations. Firstly, we included studies from only one database: PubMed, which may have led to the omission of relevant studies indexed elsewhere. However, PubMed was chosen for its comprehensive coverage of high-quality biomedical literature, making it highly relevant to our research focus. While this approach ensured relevance, future studies should consider additional databases to capture a broader range of studies. Additionally, the review was limited to English-language articles, which may overlook important studies in other languages. The reliance on peer-reviewed journal articles excluded gray literature such as conference abstracts and unpublished studies, introducing potential publication bias. All included studies were conducted by a single research group, limiting generalizability. The moderate sample sizes and geographical focus on Spain further restrict the applicability of the results. Lastly, deviations from the initial protocol, such as adjustments in the search strategy or inclusion criteria, may have influenced the findings. These limitations should be considered when interpreting the results and underscore the need for further research with more diverse and comprehensive methodologies.

## Conclusion

This scoping review highlights the potential of PE with albumin replacement as a promising treatment for AD, emphasizing the need for continued research to validate and expand upon these preliminary findings. This work lays the foundation for future investigations that could ultimately inform clinical practice and policy, aiming to improve the management and outcomes for patients with AD. Additionally, these advances contribute to the enhancement of precision and personalized medicine, offering the possibility of more tailored therapeutic approaches that align with individual patient characteristics, thereby optimizing treatment efficacy and safety in AD care (31, 32).

## Data Availability

The original contributions presented in the study are included in the article/Supplementary material, further inquiries can be directed to the corresponding author/s.
