# Corrigendum: The use of plasma exchange with albumin replacement in the management of Alzheimer's disease: a scoping review

**DOI:** 10.3389/fneur.2024.1509655

**Published:** 2024-10-29

**Authors:** Yahveth Cantero-Fortiz, Mercè Boada

**Affiliations:** ^1^Ace Alzheimer Center Barcelona, Universitat Internacional de Catalunya, Barcelona, Spain; ^2^Networking Research Center on Neurodegenerative Diseases (CIBERNED), Instituto de Salud Carlos III, Madrid, Spain

**Keywords:** Alzheimer's disease, plasma exchange, albumin replacement, cognitive function, amyloid-beta, neurodegenerative disorders

In the published article, there was an error regarding the significance of the results using “*p* < 0.05” instead of “*p* > 0.05.” Despite the Clinical relevance, there was no statistical significance.

A correction has been made to the **Results** section, subsection *Neuropsychological and quality-of-life section assessments*, paragraph 26. The sentence originally stated:

“The study employed standardized tests such as the Mini-Mental State Examination (MMSE) and the AD Disease Assessment Scale-Cognitive Subscale (ADAS-Cog) which showed statistically significant improvements (p < 0.05) in the treatment group over the 21 weeks treatment period plus 6 months follow-up.”

The correct sentence should be:

“The study employed standardized tests such as the Mini-Mental State Examination (MMSE) and the Alzheimer's Disease Assessment Scale-Cognitive Subscale (ADAS-Cog) which demonstrated clinical improvements in the treatment group. However, these improvements did not reach statistical significance (*p* > 0.05) over the 21-week treatment period plus 6 months of follow-up.”

Additionally, the term “AD disease” was used redundantly in several paragraphs.

A correction has been made throughout the article, [Paragraphs 11, 12, 14, 20, 21, 22, 26, and 35]. These sentences previously stated:

“AD disease”

The corrected sentences now state:

“AD”

The authors apologize for these errors and state that this does not change the scientific conclusions of the article in any way. The original article has been updated.

