# Supplementary material for: The use of plasma exchange with albumin replacement in the management of Alzheimer’s disease: a scoping review
Source: Front Neurol. 2024 Sep 30;15:1443132. doi: 10.3389/fneur.2024.1443132 (PMC11484623; doi:10.3389/fneur.2024.1443132)
Supplement: Supplementary file 1 [file Table_1.docx]

**Appendix 1. Protocol**

| **Section** | **Description** |
| --- | --- |
| **Introduction** | Rationale: Alzheimer's disease (AD) significantly impacts cognitive function and quality of life. Traditional treatments have limited efficacy. Plasma exchange with albumin replacement has emerged as a potential therapeutic approach. This review aims to map existing evidence, identify research gaps, and inform future research. |
| **Objectives** | To systematically map the literature on plasma exchange with albumin replacement in AD management, focusing on: Different outcomes measured; various methodologies used, reported benefits and risks. |
| **Methods** | **Eligibility Criteria:**  **Inclusion:**   - Patients diagnosed with AD - Plasma exchange with albumin replacement - Standard treatment, placebo, or other interventions - Cognitive function, brain structural changes, quality of life, and related outcomes - RCTs, longitudinal studies, observational studies, experimental Studies - Peer-reviewed journal articles   **Exclusion:**   - Review articles, editorials, opinion pieces, letters - Non-peer-reviewed articles - Studies not published in English |
| **Information Sources** | Databases:  PubMed |
| **Search Strategy** | ("Plasma exchange" OR "therapeutic plasma exchange" OR "plasmapheresis") AND ("Albumin replacement") AND ("Alzheimer’s disease") |
| **Study Selection** | Process:   - Screening Titles and Abstracts: Two independent reviewers will be screened by titles and abstracts to identify relevant studies - Exclude studies not meeting inclusion criteria - Full-Text Review: Full texts will be reviewed against criteria by two independent reviewers - Resolve discrepancies through discussion or a third reviewer |
| **Data Extraction** | A form will include the author, year of publication, study design, Population characteristics, Intervention details, comparison details, outcomes measured, Key findings, and limitations of the Study. |
| **Data Charting** | Data will be organized into tables or spreadsheets for analysis to identify themes and patterns across studies. |
| **Synthesis of Results** | Results will be synthesized narratively, highlighting key insights, themes, and gaps. Thematic analysis will be conducted to identify common outcomes and methodologies. |
| **Presentation of Results** | Findings will be presented in a structured format, including flow diagram of the study selection process, tables summarizing study characteristics and key findings, narrative summary discussing main themes, research gaps, and implications for practice and future research. |
| **Conclusion** | This Scoping review will provide a comprehensive overview of current evidence on plasma exchange with albumin replacement in AD management. Identifies gaps in the literature and suggests directions for future research. |

**Appendix 2. Search Strategy**

**Plasma Exchange with Albumin Replacement in Alzheimer's Disease – PubMed Search Strategy (Literature Search performed: May 2024)**

| 1 | Alzheimer Disease/ |
| --- | --- |
| 2 | Plasmapheresis/ |
| 3 | Plasma Exchange/ |
| 4 | ("Alzheimer's disease" and "plasmapheresis").tw. |
| 5 | ("Alzheimer's disease" and "plasma exchange").tw. |
| 6 | or/2-5 |
| 7 | 1 and 6 |
| 8 | limit 7 to english |
| 9 | limit 8 to yr=2000-current |

**Appendix 3. PRISMA** **flowchart**

**Identification of studies via databases and registers**

Records removed *before screening*:

Duplicate records removed

(n =1)

Records identified from*:

Databases (n = 40)

**Identification**

Records screened

(n = 39)

Records excluded**

(n = 25)

Reports sought for retrieval

(n = 14)

Reports not retrieved

(n =0)

**Screening**

Reports assessed for eligibility

(n = 14)

Reports excluded:

Wrong article type (n =7)

.

Studies included in review

(n = 7)

**Included**

From:  Page MJ, McKenzie JE, Bossuyt PM, Boutron I, Hoffmann TC, Mulrow CD, et al. The PRISMA 2020 statement: an updated guideline for reporting systematic reviews. BMJ 2021;372:n71. doi: 10.1136/bmj.n71. For more information, visit: <http://www.prisma-statement.org/>

**Appendix 4. Table 1 General characteristics of the included Studies**

| **Study ID (Author, year)** | **Study Design** | **Country** | **Setting** | **Participants (n)** | **Participants Age** | **Sex Distribution** | **Interventions** | **Comparisons** | **Outcomes** |
| --- | --- | --- | --- | --- | --- | --- | --- | --- | --- |
| Boada et al., 2017 | RCT | Spain | Clinical and hospital settings | 42 | Mean age 74.6 years | 57% female | Plasma exchange with 5% albumin | Placebo | Cognitive function, CSF and plasma amyloid-β concentrations |
| Boada et al., 2019 | RCT | Spain | Clinical and hospital settings | 347 | Mean age 73.8 years | 60% female | Plasma exchange with albumin replacement | Standard care | Cognitive function, Quality of life |
| Boada et al., 2020 | RCT | Spain | Clinical and hospital settings | 347 | Mean age 75.1 years | 58% female | Plasma exchange with albumin replacement | Standard care | Cognitive function, Disease progression, Quality of life |
| Boada et al., 2022 | RCT | Spain | Clinical and hospital settings | 120 | Mean age 72.3 years | 56% female | Plasma exchange with albumin replacement | Placebo | Cognitive function, Neuropsychiatric outcomes, Quality of life |
| Boada et al., 2023 | RCT | Spain | Clinical and hospital settings | 322 | Mean age 69.0 years | 54% female | Plasma exchange with albumin and IVIG | Placebo | Neuroimaging outcomes, Cognitive function, Metabolic activity |
| Cuberas-Borrós et al., 2018 | RCT | Spain | Clinical and hospital settings | 42 | Mean age 74.1 years | 59% female | Plasma exchange with 5% albumin | Placebo | Brain perfusion, Hippocampal volume |
| Cuberas-Borrós et al., 2022 | RCT | Spain | Clinical and hospital settings | 213 | Mean age 72.3 years | 56% female | Plasma exchange with albumin and IVIG | Placebo | Brain metabolism, Structural brain changes |

**Appendix. 5**

**Table 2. Cochrane Risk of Bias Tool**

| **Domain** | **Boada 2017** | **Cuberas-Borrós 2018** | **Boada 2019** | **Boada 2020** | **Cuberas-Borrós 2022** | **Boada 2022** | **Boada 2023** |
| --- | --- | --- | --- | --- | --- | --- | --- |
| **Random Sequence Generation** | Low | Low | Low | Low | Low | Low | Low |
| **Allocation Concealment** | Low | Low | Low | Low | Low | Low | Low |
| **Blinding of Participants and Personnel** | Low | Low | Low | Low | Low | Low | Low |
| **Blinding of Outcome Assessment** | Low | Low | Low | Low | Low | Low | Low |
| **Incomplete Outcome Data** | Low | Low | Low | Low | Low | Low | Low |
| **Selective Reporting** | Low | Low | Low | Low | Low | Low | Low |
| **Other Sources of Bias** | Unclear | Unclear | Unclear | Unclear | Unclear | Unclear | Unclear |
| **Overall Score** | Low | Low | Low | Low | Low | Low | Low |

**Appendix 6. Excluded Studies**

| **Title** | **Authors** | **Year** | **Reason for Exclusion** |
| --- | --- | --- | --- |
| Treatment of Alzheimer disease using combination therapy with plasma exchange and haemapheresis with albumin and intravenous immunoglobulin: Rationale and treatment approach of the AMBAR (Alzheimer Management By Albumin Replacement) study | Boada et al. | 2016 | Review article |
| Therapeutic Plasmapheresis with Albumin Replacement in Alzheimer's Disease and Chronic Progressive Multiple Sclerosis: A Review | Navarro-Martínez et al. | 2020 | Review Article |
| AMBAR, an Encouraging Alzheimer's Trial That Raises Questions | Loeffler | 2020 | Review Article |
| Emerging insights into the role of albumin with plasma exchange in Alzheimer's disease management | Costa et al. | 2021 | Review Article |
| Therapeutic plasma exchange with albumin: a new approach to treat Alzheimer's disease | Boada et al. | 2021 | Review Article |
| Plasma exchange with albumin replacement and disease progression in amyotrophic lateral sclerosis: a pilot study | Povedano et al. | 2022 | Not Related to Alzheimer's Disease |
| Plasma Exchange in Alzheimer's Disease | Rohrer et al. | 2023 | Review Article |

**Principio del formulario**
